# Proposal for a Global Classification and Nomenclature System for A/H9 Influenza Viruses

**DOI:** 10.3201/eid3008.231176

**Published:** 2024-08

**Authors:** Alice Fusaro, Juan Pu, Yong Zhou, Lu Lu, Luca Tassoni, Yu Lan, Tommy Tsan-Yuk Lam, Zoe Song, Justin Bahl, Jiani Chen, George F. Gao, Isabella Monne, Jinhua Liu, The International H9 Evolution Consortium

**Affiliations:** Istituto Zooprofilattico Sperimentale delle Venezie, Legnaro, Italy (A. Fusaro, L. Tassoni, I. Monne);; China Agricultural University, Beijing, China (J. Pu, Y. Zhou, J. Liu);; University of Edinburgh, Edinburgh, Scotland, UK (L. Lu);; Chinese Center for Disease Control and Prevention, Beijing (Y. Lan);; The University of Hong Kong, Hong Kong, China (T.T.-Y. Lam, Z. Song);; University of Georgia, Athens, Georgia, USA (J. Bahl, J. Chen);; Chinese Academy of Sciences, Beijing (G.F. Gao)

**Keywords:** A/H9 influenza viruses, influenza, hemagglutinin, phylogeny, classification, nomenclature, global, viruses, respiratory infections, *Suggested citation for this article*: Fusaro A, Pu J, Zhou Y, Lu L, Tassoni L, Lan Y, et al. Proposal for a global classification and nomenclature system for A/H9 influenza viruses. Emerg Infect Dis. 2024 August [*date cited*]. https://doi.org/10.3201/eid3008.231176

## Abstract

Influenza A/H9 viruses circulate worldwide in wild and domestic avian species, continuing to evolve and posing a zoonotic risk. A substantial increase in human infections with A/H9N2 subtype avian influenza viruses (AIVs) and the emergence of novel reassortants carrying A/H9N2-origin internal genes has occurred in recent years. Different names have been used to describe the circulating and emerging A/H9 lineages. To address this issue, an international group of experts from animal and public health laboratories, endorsed by the WOAH/FAO Network of Expertise on Animal Influenza, has created a practical lineage classification and nomenclature system based on the analysis of 10,638 hemagglutinin sequences from A/H9 AIVs sampled worldwide. This system incorporates phylogenetic relationships and epidemiologic characteristics designed to trace emerging and circulating lineages and clades. To aid in lineage and clade assignment, an online tool has been created. This proposed classification enables rapid comprehension of the global spread and evolution of A/H9 AIVs.

Avian influenza viruses (AIVs), including type A of the H9 subtype (A/H9), have a worldwide distribution in wild birds and are endemic in poultry populations in several countries in Asia, the Middle East, and Africa. Despite being classified as low pathogenicity, AIVs, strains of the A/H9N2 subtype are becoming an increased threat to domestic birds and humans (*1*,*2*). A/H9N2 subtype viruses can cause major economic damage to the poultry industry because of decreased egg production and increased mortality, related to co-infection with bacteria and other viruses (*3*–*6*). The economic consequences from this subtype have prompted several countries to adopt poultry vaccination policies to try to control the virus spread (*7*,*8*). Those policies have further driven genetic diversification of A/H9 AIVs (*8*–*11*).

Since 1998, viruses of the A/H9 subtype have occasionally crossed over into mammal species, leading to detection in humans, pigs, dogs, horses, pikas, minks, and bats (*12*). As of December 1, 2023, A/H9N2 was associated with 128 human infections, 90% of which were reported in China (*13*), and more than half of cases (52%) were identified during 2020–2023. Of note, this number may be underestimated because A/H9N2 infected humans do not often show obvious symptoms (*14*). Furthermore, A/H9N2 AIVs were identified as a source of internal gene mutations, leading to the emergence of several reassortants in human infection reports, such as H3N8, H5N6, H7N9, and H10N8 (*15*–*21*).

To date, different names have been adopted to describe A/H9 influenza virus lineages or sublineages and clades based on the hemagglutinin (HA) gene, often with differing names for the same group (*5*,*22*–*27*). The most widely accepted 4 primary lineages have multiple and complex names: the BJ/94 lineage is also known as Y280-like, G9-like, or h9.4.2 (represented by A/chicken/Beijing/1/94, A/chicken/Hong Kong/G9/97, or A/duck/Hong Kong/Y280/97); the G1 lineage is also known as h9.4.1 (represented by A/quail/Hong Kong/G1/97); the Y439 lineage is also known as Korean or h9.3 (represented by A/chicken/Korea/38349-p96323/96 or A/duck/Hong Kong/Y439/97); and the American lineage is also known as h9.1-h9.2 (represented by A/turkey/Wisconsin/1/1966 or A/turkey/Minnesota/511/1978). Additional sublineages and clades continue to emerge because of the rapid evolution of the A/H9 virus (*8*,*22*,*23*,*26*–*29*). The difference in clade classification and naming hinders comparison between studies and challenges critical discussion on the epidemiology, evolution, and spread of this subtype.

To address this issue, an international consortium of scientists from 22 laboratories in Europe, Asia, Africa, and the Americas was established to create a uniform system for classifying A/H9 viruses. This group was endorsed by the WOAH/FAO (World Organisation for Animal Health/Food and Agriculture Organization of the United Nations) Network of Expertise on Animal Influenza. By reconstructing a comprehensive phylogenetic history of all globally collected and publicly available HA sequences of A/H9 viruses, of any neuraminidase subtype, the consortium worked to create a harmonized classification and nomenclature system for A/H9 to be used in future epidemiologic and evolutionary studies.

## Methods

### Sequence Data and Metadata Preparation

To provide a comprehensive picture of A/H9 genetic diversity, we generated a dataset of the HA gene that included every H9-HA sequence available on the GISAID (https://www.gisaid.org) and GenBank public databases, which we accessed on July 18, 2022. We aligned the sequence dataset by using MAFFT v7.0 (*30*) and curated as described (Appendix 1). A final dataset containing 10,638 HA sequences and the related information, including accession numbers, resulted after our quality check process (Appendix 2 Table 1).

### Phylogenetic Characterization

**Figure 1:**
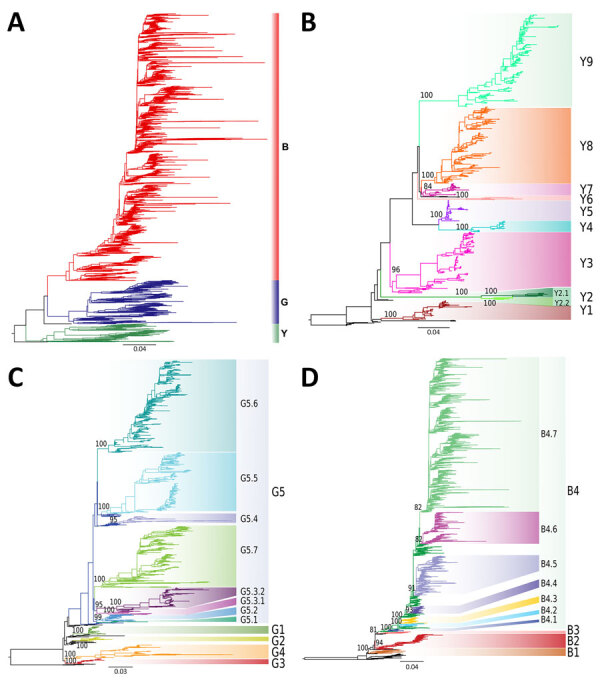
Phylogenetic trees and assigned clades of as part of a proposed global classification and nomenclature system for A/H9 influenza viruses. A) Maximum-likelihood (ML) phylogenetic tree obtained using the complete dataset. The branches are colored according to the 3 identified lineages. A/quail/Hong Kong/A28945/1988 and A/Quail/Hong Kong/AF157/1992 were used as an outgroup black). B) ML phylogenetic tree of the Y lineage dataset. Six sequences of the G lineage were used as the outgroup. Numbers next to the clade-defining nodes represent ultra-fast bootstrap supports. Clades are labeled and marked in colors. C) ML phylogenetic tree of the G lineage. Six sequences of the Y lineage were selected as the outgroup. Numbers next to the clade-defining nodes represent ultra-fast bootstrap supports. Clades are labeled and marked in colors. D) ML phylogenetic tree of the B lineage. Six sequences of the Y lineage were used as the outgroup. Numbers next to the clade-defining nodes represent ultra-fast bootstrap supports. Clades are labeled and marked in colors. Scale bars indicate substitutions per site.

We generated a maximum-likelihood (ML) phylogenetic tree from the final complete dataset (n = 10,638) of the A/H9 virus HA genes by using IQ-TREE v1.6 (*31*) and applying the best-fitted nucleotide substitution model selected by ModelFinder (*32*). We performed clustering of the ML phylogenies of the complete dataset by using the pathogen-agnostic clustering tool PhyCLIP (Appendix 1 Figure 1) (*33*).

We conducted validation of lineages and clades identified with PhyCLIP by using multiple datasets and software to perform phylogenetic analyses. We assessed the robustness of our inference on a down-sampled dataset of 1,000 sequences, and then we assessed the nodal supports on a down-sampled dataset of 2,000 sequences. We selected sequences on the basis of phylogenetic diversity and calculated them as the sum of branch lengths of the minimal subtree spanned by a set of taxa included in the phylogenetic tree. We conducted this selection by using the phylogenetic diversity analyzer tool (*34*), starting from the ML tree obtained from the complete dataset. We generated ML trees from the 2 down-sampled datasets in IQ-TREE v2.1.3 by performing ultrafast-bootstrap resampling analysis (1,000 replications) (*31*,*35*). We used the best-fitted nucleotide substitution model selected by ModelFinder, implemented in IQ-TREE (*32*). We analyzed lineages separately to characterize the different clades within each lineage. We generated 3 datasets: Y_dataset, G_dataset, and B_dataset.

To assess the consistency of the clades, we conducted phylogenetic analyses for each dataset by using IQ-TREE v 2.1.3 (*31*) and PhyML v3.0 (*36*). We used ModelFinder, implemented in IQ-TREE (*32*), to select the best-fit nucleotide substitution model for each dataset. We assessed nodal supports in the IQ-TREE and PhyML analyses by using ultrafast-bootstrap and Shimodaira-Hasegawa (SH)–like branch supports (*35*,*36*).

To visualize the phylogenetic trees, we used FigTree version 1.4.2 (Figtree, http://tree.bio.ed.ac.uk/software/figtree). We calculated within and between clades average pairwise nucleotide distances (APD) by using MEGA X (p-distance) (*37*). We used TreeTime (https://treetime.readthedocs.io/en/latest) to reconstruct the ancestral sequence, which enabled us to infer the nonsynonymous nucleotide mutations leading to amino acid variations at each internal node (*38*).

### Pilot Dataset

After the clade identification process, we created subsets for each lineage. The subsets are pilot_Y (86 sequences), pilot_G (105 sequences), and pilot_B (101 sequences); pilot_complete included all 3 lineages (292 sequences) (Appendix 3). We created the 4 subsets by using the phylogenetic diversity analyzer tool (*34*) and then manually selected sequences of underrepresented lineages and clades (Appendix 3). We implemented ML phylogenetic analyses with 1,000 ultrafastbootstrap and 1,000 nonparametric bootstrap replicates to ensure that the topology inferred from the larger datasets would be maintained with fewer sequences.

### Online Tool Development

We created our online tool to automate the lineage classification of A/H9 influenza viruses. For the tool creation, we used Python (Python, https://www.python.org) and added an interactive display through the Django framework (Django, https://www.djangoproject.com). The tool assesses user-provided sequences to determine if they belong to the A/H9 subtype. If confirmed, the tool further classifies the sequences into their specific lineage and clade. The query sequences will be included in a pilot dataset, and a ML phylogenetic tree will be constructed by using IQ-TREE. The output from the tool includes the clade annotation and ML phylogenetic tree.

## Results

### Principles of the Nomenclature System

The genomic characterization of A/H9 is needed to support the surveillance, prevention, and risk assessment of this globally prevalent subtype. A unified criterion for A/H9 genotyping is necessary to compare molecular epidemiology data obtained from different laboratories. We propose a workable and practical lineage classification and nomenclature for A/H9 AIVs on the basis of a comprehensive phylogenetic analysis of all available HA gene sequences. Our proposed nomenclature of A/H9 AIVs meets multiple requirements by capturing local and global patterns of virus genetic diversity in a timely and coherent manner, being robust and flexible enough to accommodate emerging virus diversity, providing support to track emerging clades when they exhibit amino acid or biologic and epidemiologic changes, and being informative and easily traceable while enabling the incorporation of the major and minor clades over time.

### Clade Classification of A/H9 Viruses

**Figure 2:**
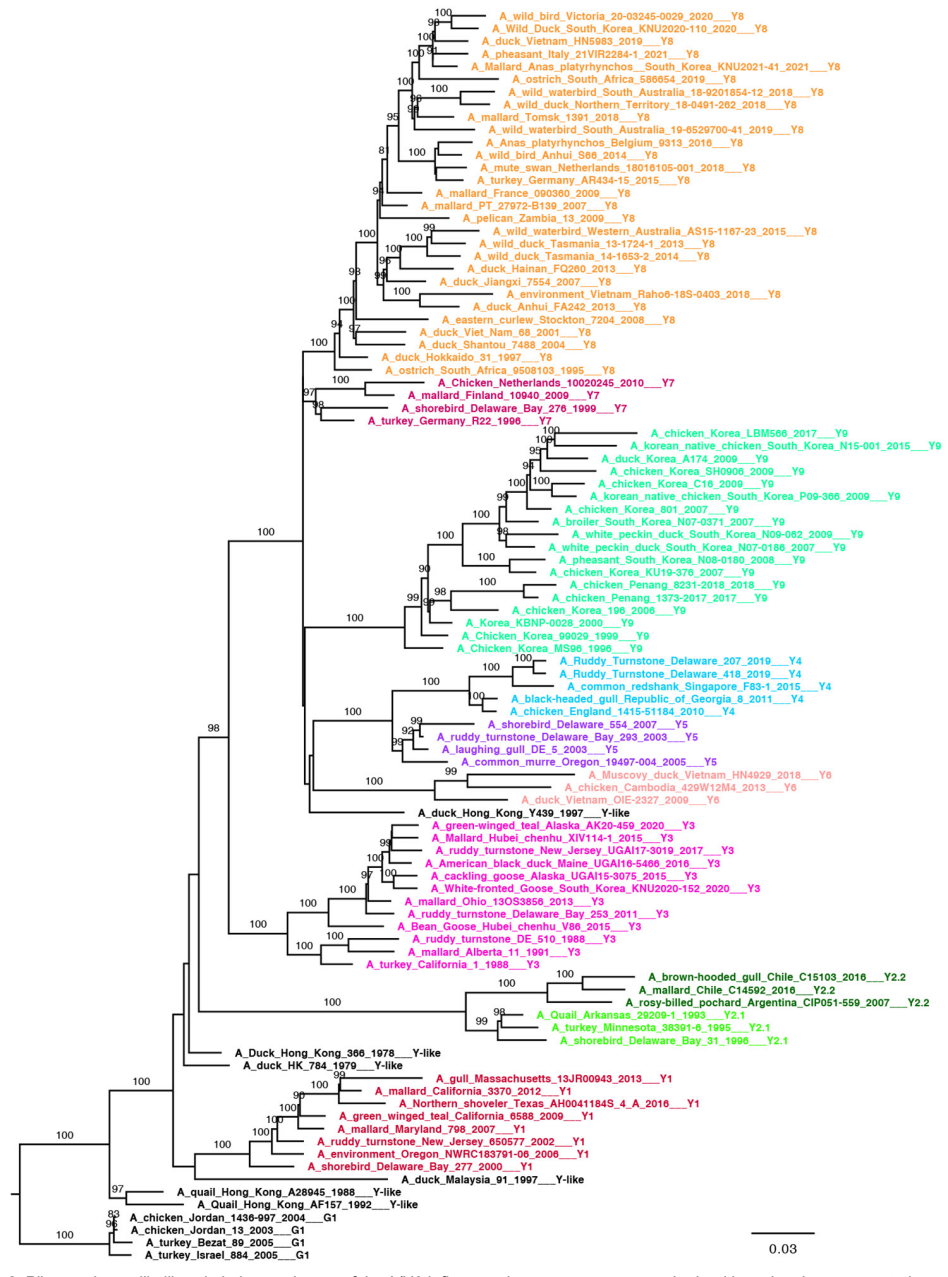
Pilot maximum-likelihood phylogenetic tree of the A/H9 influenza virus gene sequences obtained by using the representative dataset (Appendix 3) for the Y lineage provided as part of a proposed global classification and nomenclature system for A/H9 influenza viruses. Each clade is represented by >3 sequences, each labeled and colored according to the clade of belonging. Ultrafast-bootstrap supports >80% are indicated next to nodes. Scale bar indicates substitutions per site.

We used a curated dataset of 10,638 global A/H9 HA sequences (Appendix 2 Table 1) to assess the phylogenetic relationships among globally circulating A/H9 viruses and establish a unified classification system for the virus. We applied 5 steps to assign and identify clades (Appendix 1 Figure 2) by integrating phylogenetic topology, branch support, genetic distances, epidemiologic information, and amino acid variants shared within each group. We defined clades on the basis of the following criteria: clades are assigned to monophyletic groups including >3 sequences obtained from viruses sampled over a period >3 years, indicating sustained transmission of the clade in >2 influenza seasons; clades have an ultrafast-bootstrap value >80% for the clade-defining node; HA proteins within the clade must share >1 amino acid mutation; an APD of >6% between each clade is recommended. However, to meet all other criteria, the APD may be slightly lower for certain clades.

Using those criteria, we defined 3 lineages, 18 first-order clades, 16 second-order clades, and 2 third-order clades (Figure 1). Nodal supports for each clade obtained from different analysis methods of each complete or down-sampled datasets are available (Appendix 1 Table 1). Each clade contains >1 amino acid change at the internal nodes (Appendix 1 Table 2). We found many mutations associated with host tropism, virulence, or antigenicity of the influenza virus (*8*), indicating that A/H9 AIVs of these clades may have undergone changes in biologic characteristics.

### Nomenclature Criteria of A/H9 Lineages and Clades

To maintain consistency with traditional and commonly used lineage names for A/H9 (Y439, American, G1, and BJ/94 lineages) that convey information on spatial and biologic characteristics, we opted to label the 3 lineages as Y (to correspond with Y439 and American lineages), G (to correspond with G1 lineages), and B (to correspond with BJ/94 lineages). The numeric values, such as Y1 or G1 for the first-order clade, Y1.1 or G1.1 for the second-order clade, Y1.1.1 or G1.1.1 for the third-order clade, are used to identify clades that descended from the Y, G, and B lineages. In addition, viruses are classified as Y-like, G-like, or B-like if they belong to 1 of the identified lineages but do not fall within any clade. We provided a comparison between our proposed nomenclature system and some of the previously used A/H9 nomenclatures that enables rapid mapping of our lineages and clades to the name of the previously used lineages and clades (Appendix 1 Table 3).

### Pilot Datasets of Lineages and Clades

**Figure 3:**
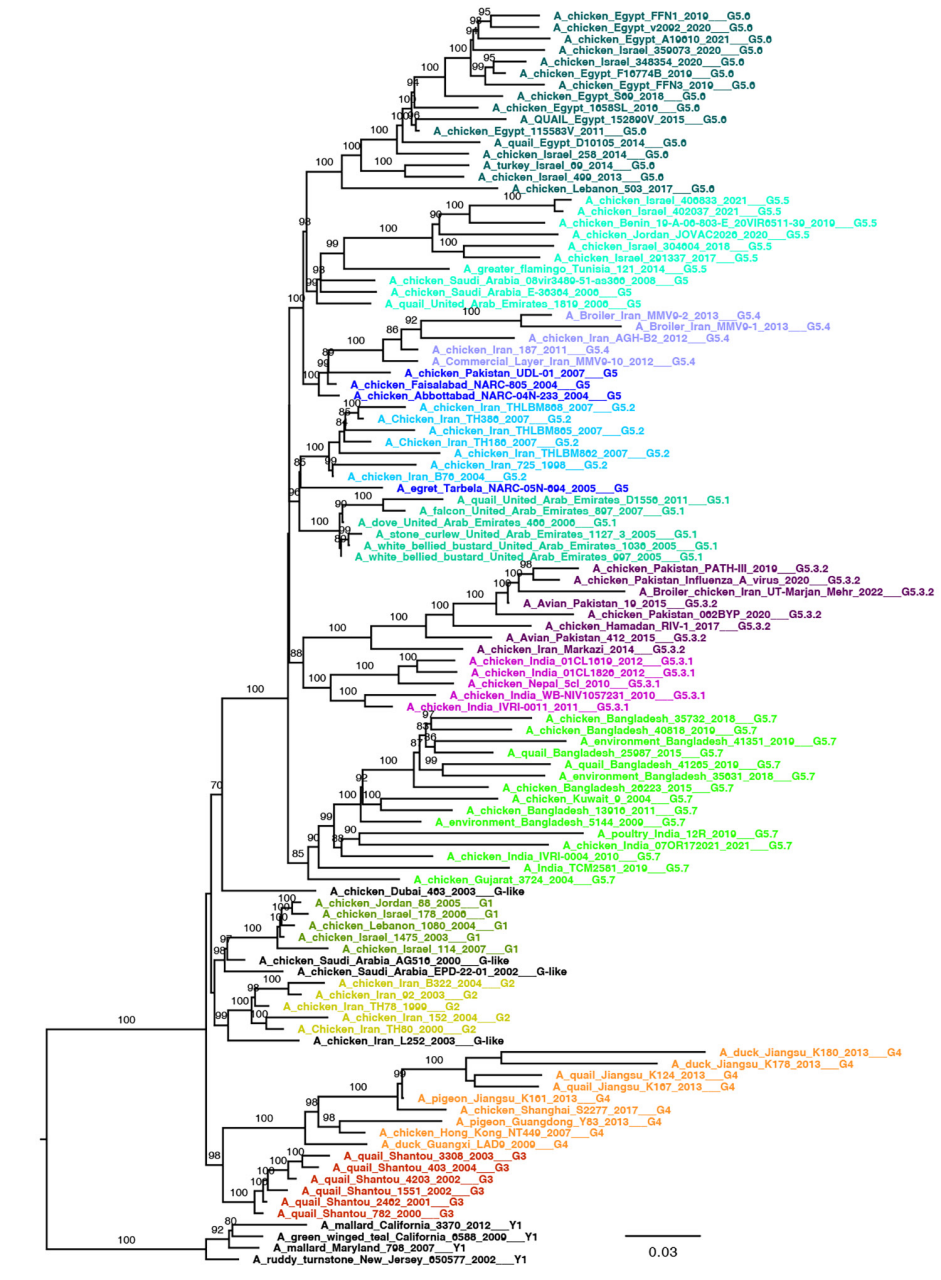
Pilot maximum-likelihood phylogenetic tree of the A/H9 influenza virus gene sequences obtained by using the representative dataset (Appendix 3) for the G lineage provided as part of a proposed global classification and nomenclature system for A/H9 influenza viruses. Each clade is represented by >3 sequences, each labeled and colored according to the clade of belonging. Ultrafast-bootstrap supports >80% are indicated next to nodes. Scale bar indicates substitutions per site.

**Figure 4:**
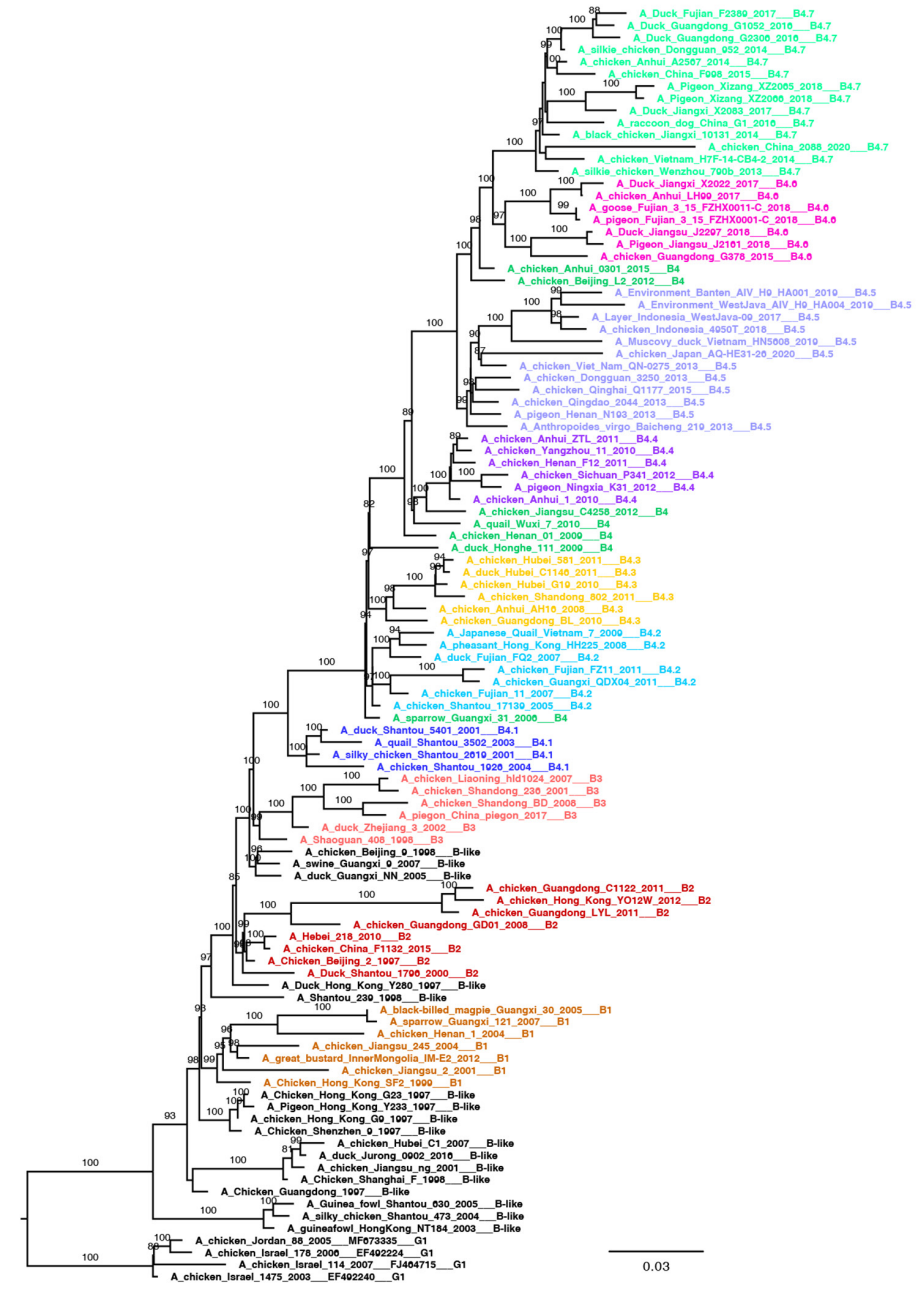
Pilot maximum-likelihood phylogenetic tree of the A/H9 influenza virus gene sequences obtained by using the representative dataset (Appendix 3) for the B lineage provided as part of a proposed global classification and nomenclature system for A/H9 influenza viruses. Each clade is represented by >3 sequences, each labeled and colored according to the clade of belonging. Ultrafast-bootstrap supports >80% are indicated next to nodes. Scale bar indicates substitutions per site.

We generated 3 small datasets of ≈100 sequences containing representative viruses for each clade of the Y, G, and B lineages. To ensure that topology is maintained with a lower number of sequences, we performed ML phylogenetic reconstructions. All lineage and clade-defining branches are strongly supported (Appendix 1 Table 1) and the topologies of the generated pilot trees (Figure 2; Figure 3; Figure 4; Appendix 1 Figure 4) are consistent with the trees generated from larger datasets. All pilot datasets that contain sequences labeled according to lineage and clade are available (Appendix 3). Those pilot datasets can be used for rapid classification of newly sequenced A/H9 viruses according to our proposed classification scheme. If the lineage is unknown, the nucleotide A/H9 HA gene sequence or sequences under investigation can be analyzed by using the complete pilot dataset that includes all 3 lineages (Appendix 3). The complete pilot dataset will identify both the lineage and the specific clade according to clustering. If the lineage is known, the phylogenetic analysis can be performed by using the small and easily manageable pilot dataset specific for the lineage of interest, either pilot G, pilot Y, or pilot B (Appendix 3). The lineage and clade of an H9 sequence can also be identified by using the online tool.

### Genetic and Epidemiologic Characteristics of Individual A/H9 Lineages and Clades

To determine whether the nomenclature system can reflect the real epidemiologic characteristics of A/H9 influenza viruses, we compiled a detailed description for those lineages and clades (Appendix 1 Tables 4–6; Appendix 2 Table 2) that includes the temporal, spatial, and host distribution. However, considering the limited number of sequences available for certain countries and the limited sampling data from wild birds, sampling bias cannot be excluded.

### Y Lineage

**Figure 5:**
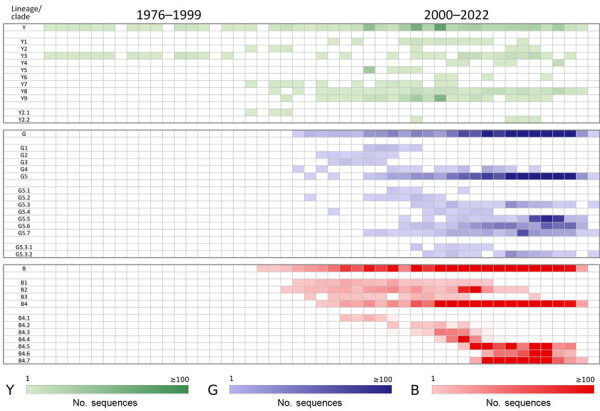
Temporal distribution of each lineage and clade for A/H9 influenza viruses as part of a proposed global classification and nomenclature system for A/H9 influenza viruses. The heat map displays the number of sequences for each lineage and clade per year.

**Figure 6:**
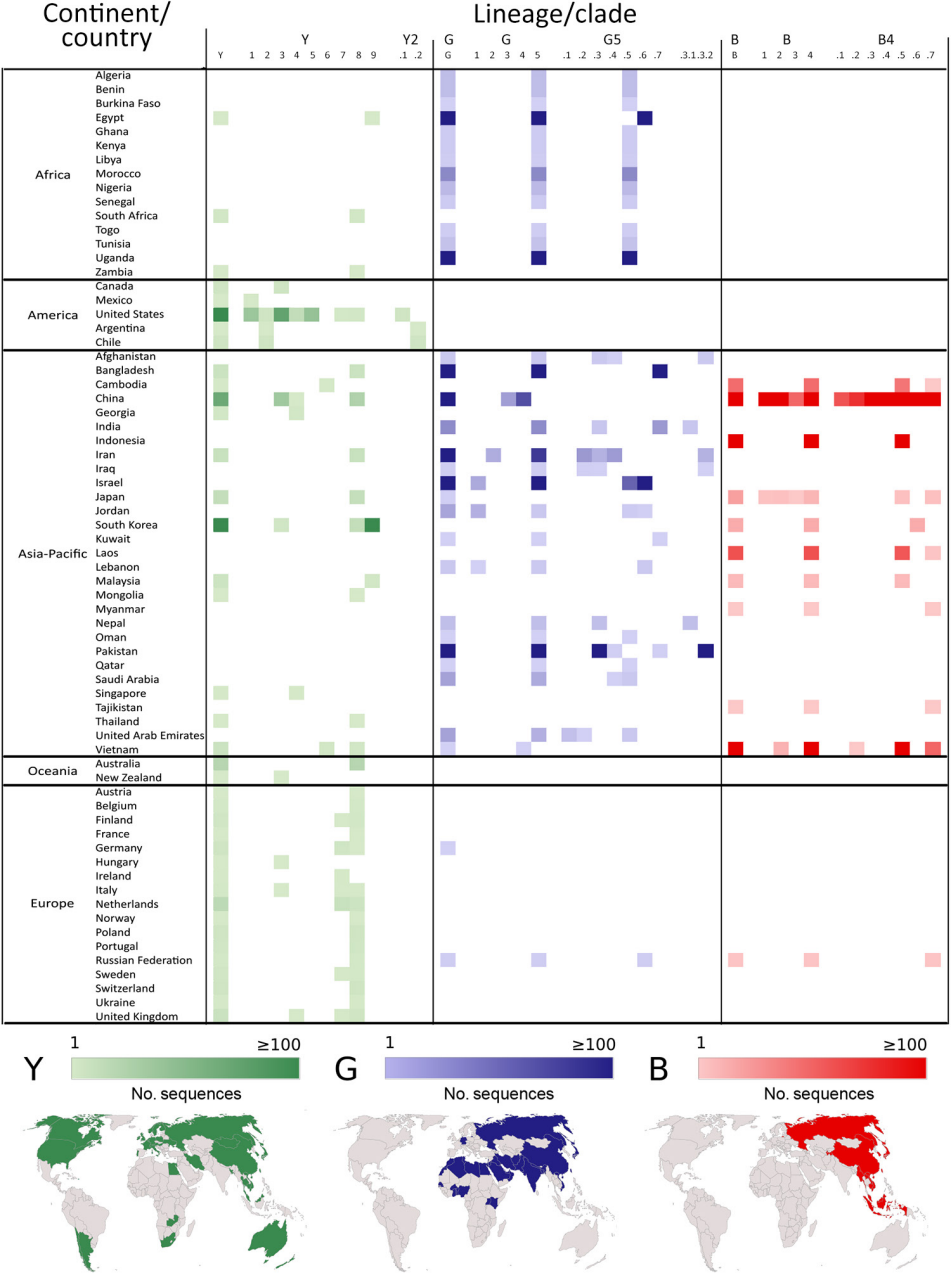
Geographic distribution of each lineage and clade for A/H9 influenza viruses as part of a proposed global classification and nomenclature system for A/H9 influenza viruses. The heat map displays the number of sequences for each lineage and clade per country. Countries were ordered by their macro-region (upper panels). Each country displaying >1 sequence was colored on the map in green (Y lineage), blue (G lineage), or red (B lineage) (bottom panels).

The Y lineage (n = 622), including the previously named Y439 (or Korean, h9.3) and American (or h9.1–h9.2) lineages (*27*,*39*), was first identified in 1966 and is the oldest and most widespread lineage of A/H9 influenza viruses (*40*). In our dataset, the group contains sequences identified after 1976 because we removed earlier strains such as A/turkey/Wisconsin/1/1966 (Figure 5; Appendix 1 Table 4). The Y lineage has spread to every continent (Figure 6; Appendix 1 Table 4) and has evolved into 9 first-order (Y1–Y9) and 2 second-order (Y2.1 and Y2.2) well-supported clades (ultrafast-bootstrap >84, SH-like >0.85) (Figure 1, panel B). Clades of the Y lineage exhibited a high between-clade APD ranging from 7.10% to 18.30% (Appendix 1 Figure 3; Appendix 2 Table 2). Clades Y2.1 (South America, 2007–2017) and Y2.2 (United States, 1993–1996) showed the highest genetic distance from all other clades (15.37%–18.30%). Within-clade APD was <6% for all Y lineage clades, ranging from clade Y5 at 1.19% (United States, 2005–2007) to clade Y6 at 5.71% (Cambodia, Vietnam, 2009–2018).

**Figure 7:**
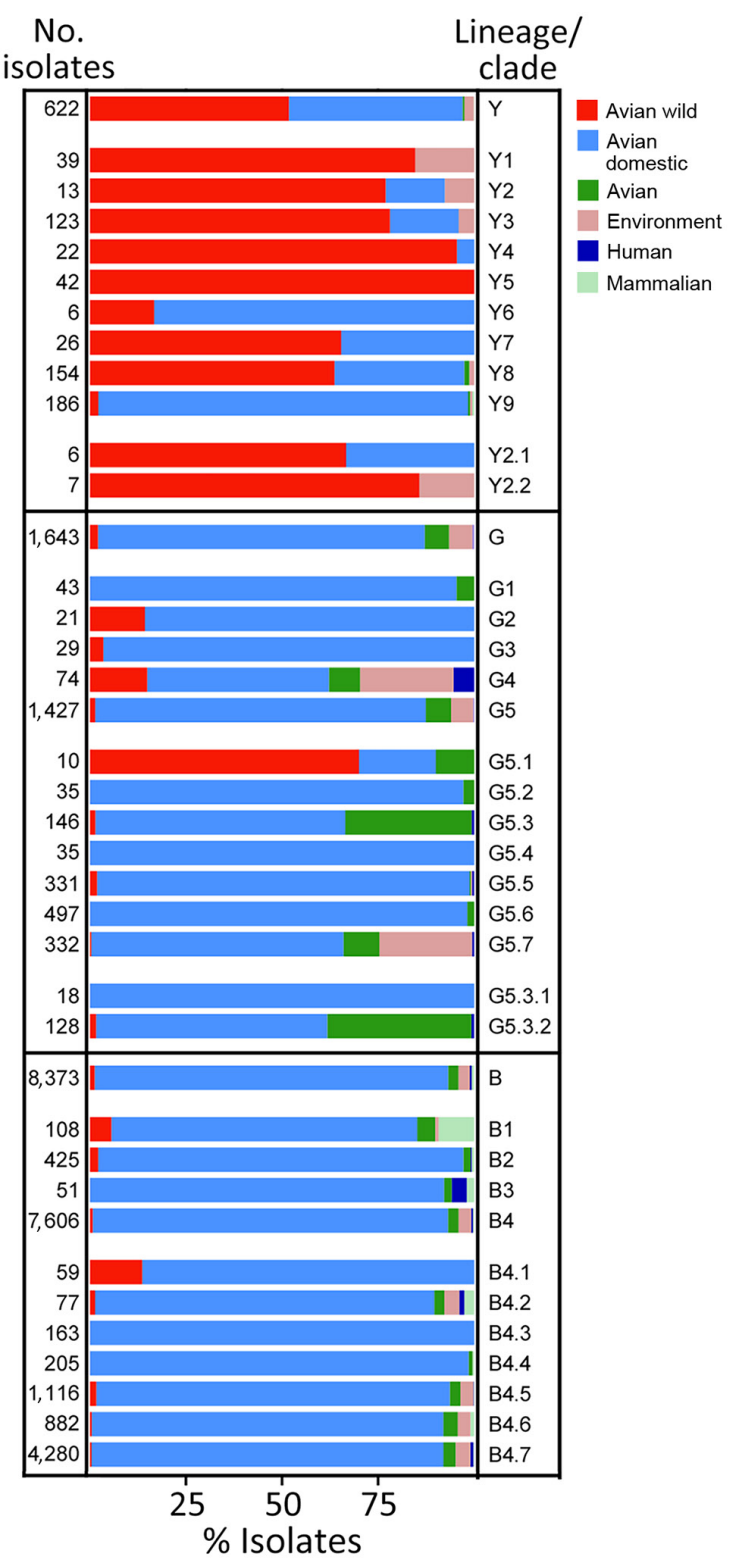
Host distribution of each lineage and clade for A/H9 influenza viruses as part of a proposed global classification and nomenclature system for A/H9 influenza viruses. The bar chart illustrates the percentage of host composition for each lineage and clade. Hosts are grouped into the following categories, represented as colors on each bar: avian wild, avian domestic, avian (birds that are not identified as wild or domestic), environment, human, and mammalian (other than human).

Clades Y3, Y4, Y7, and Y8 were detected in >3 countries on >2 different continents, and clade Y8 has the widest geographic distribution: Australia, Asia, Europe, Africa, and North America. The remaining clades were identified in fewer countries. Clades Y1, Y2, and Y5 are exclusively from the Americas, and clade Y6 is specific to Southeast Asia (Cambodia and Vietnam). Detections of Y lineage viruses are equally distributed between wild and domestic birds, and 1 detection occurred in a mammal host (swine). The proportion of wild and domestic hosts varies across the identified clades. Domestic poultry accounts for 83% of clade Y6 detections and 96% of clade Y9 detections, whereas the other clades have been identified from mostly wild birds (64%–100%) (Figure 7; Appendix 1 Table 4).

### G Lineage

The G lineage (n = 1643), previously known as G1 or h9.4.1 (*27*,*39*), has been circulating since 1997 (Figure 5; Appendix 1 Table 5). Domestic birds account for 85% of G lineage sequences (Figure 7), and the lineage is found in countries in Asia (61%) and Africa (38%) (Figure 6; Appendix 1 Table 5). According to the proposed clade classification criteria, we distinguished 5 first-order clades (G1–G5). Between-clade APD of the G lineage ranges from 4.81% to 13.59% (Appendix 1 Figure 3; Appendix 2 Table 2). Clade G5 shows the highest genetic distance from the other clades (10.94%–13.59%). Clade G5 was detected in 29 countries and was further divided into second-order (G5.1–G5.7) and third-order (G5.3.1–G5.3.2) subclades (Figure 1, panel C). The genetic distance between G1 and G2 is 5.81% and between G5.1 and G5.2 is 4.81%; those distances are less than the established 6% cutoff but are considered acceptable because they do not share a stable common root node. Within-clade APD was <6% for all clades, ranging from clade G1 (Israel, Jordan, Lebanon, 2003–2007) at 1.30% to clade G5.7 at 5.84% (Bangladesh, India, Kuwait, Pakistan, 2003–2022) (Appendix 1 Figure 3; Appendix 2 Table 2). All the identified clades are well supported (ultrafast-bootstrap >95; SH-like >0.73).

Most of the G clade viruses originate from domestic poultry, except for G5.1, which was found in 70% of the viruses sampled from wild avian species (Figure 7; Appendix 1 Table 5). Since 1999, human infections of viruses belonging to clades G4 (n = 4, 1999–2009), G5.3.2 (n = 1, 2015), G5.5 (n = 2, 2019), and G5.7 (n = 2, 2011–2019) have been detected.

### B Lineage

The B lineage (n = 8,373), previously known as BJ/94, Y280, G9, or h9.4.2 (*27*,*39*), has been circulating since 1994 in domestic birds (92% of the sequences) from Asia, and >93% of the sequenced viruses originated from China (Figure 5; Figure 6; Figure 7; Appendix 1 Table 6). We divided lineage B into 11 well supported (ultrafast-bootstrap >81; SH-like >0.9) clades, consisting of 4 first-order clades (B1–B4) and 7 second-order clades (B4.1–B4.7) (Figure 1, panel D). Compared with the other lineages, clades of the B lineage exhibited lower between-clade APD, ranging from 4.48% to 12.06% (Appendix 1 Figure 3; Appendix 2 Table 2). Clade B4 shows the highest genetic distance from all the other clades (10.54%–12.06%). Clade B4 has had a considerable geographic expansion in the past 10 years. This clade affects 11 distinct countries in Asia and has further diversified into 7 second-order clades (B4.1–B4.7). As with the G lineage, the B lineage displayed a genetic distance between some clades of <6%. Within-clade APD was <6% for all clades, ranging from clade B4.4 at 2.18% (China, 2009–2020) to clade B4.7 at 5.04% (Cambodia, China, Japan, Laos, Myanmar, Russian Federation, Tajikistan, Vietnam, 2012–2021) (Figure 6; Appendix 1 Table 6; Appendix 2 Table 2).

Within the B lineage, all viruses sequenced in the past 5 years fall into clades B4.4–B4.7. Most of the viruses in each B clade were identified in poultry (Figure 7; Appendix 1 Table 6), and almost all clades contain viruses collected from mammal species. Most viruses recovered from human infections (82.9%, 34/41) fall within clade B4.7 (2014–2021).

### Online Tool for Automatic Sequence Classification

To enhance the accessibility of our A/H9 influenza virus clade classification and nomenclature system, we developed an intuitive online tool (https://nmdc.cn/influvar/tools/H9aiv) with the aim to provide a user-friendly interface, making it easier for researchers and stakeholders to use and navigate the classification system. Users will be able to access the website and submit their A/H9 sequence file in the appropriate format. The tool will assign the corresponding clade and will provide a phylogenetic tree. 

## Discussion

Establishing a unified nomenclature system for A/H9 influenza viruses is essential for the provision of communication to address the increasing global threat of the viruses. This system is characterized by 4 key hallmarks: comprehensiveness, robustness, inclusiveness, and practicality.

For comprehensiveness, we have created a system based on the HA gene sequence data of all the A/H9 viruses (including different neuraminidase subtypes) collected worldwide during 1976–2022. Those data enable understanding of the complete evolution of A/H9 AIVs on a regional and global scale, as well as the pattern of geographic and host distribution of lineages and clades. For example, the G and Y lineages are found in multiple continents, indicating a wide geographic diffusion, whereas the B lineage is isolated primarily in Asia and has occasional spillover to Europe through Russia. In addition, we present a record of all A/H9 AIVs clades, past and presently circulating, to provide a comprehensive understanding of the virus evolution trajectory in different host and geographic contexts.

Our system demonstrates robustness by being built from phylogenetic analysis of high-quality, nearly complete (>75%) HA gene sequences from multiple datasets (complete and down-sampled) and software packages. We have included all unique publicly available sequences, from a wide variety of origins and collection dates, to ensure the comprehensiveness and representativeness of our dataset. We only accepted high-supported clades consistently recognized across all trees, regardless of the datasets and approaches used, and characterized by a specific set of shared amino acid mutations. For example, there are 4 widely and internationally recognized lineages of A/H9 viruses, American, Y439, G1, and BJ/94 (Y280). However, because our classification approach revealed that the HA sequences of American-like and Y439-like lineages are phylogenetically closely related and share an ancestral node, they were combined into a single lineage and renamed as the Y lineage.

The inclusiveness of our system stems from the establishment of multiple criteria to identify and classify novel clades that emerge, incorporating phylogenetic evidence, molecular evidence, and epidemiologic features. Accommodating all the criteria can be challenging, because natural virus evolution is unlikely to always produce discrete boundaries. Among the criteria, the phylogenetic relationship and epidemiologic characteristics are the most useful for classification, whereas a certain degree of flexibility can be acceptable for bootstrap or APD value, provided there is strong evidence that the monophyletic clade exists and is epidemiologically relevant. This flexibility should enable the monitoring of ongoing epidemics in that clade, especially the emergence and evolution of antigenically distinct strains. The balance between the fixed criteria and topology or epidemiologic characters is also applied in the classification of H5 influenza viruses and SARS-CoV-2 (*41*,*42*).

Practicality is the most relevant hallmark of our system. We have fully considered the needs of users and have made major efforts to achieve full applicability of our system. Users can follow simple and clear criteria to identify novel clades, understand the biologic and epidemiologic significance of clades, and communicate results of their investigations through an informative nomenclature system. In assigning lineage names, we considered the traditional nomenclature in use to create consistency with historic classifications and to enable comparisons with previous studies.

In summary, our proposed nomenclature system and online tool for A/H9 AIVs is valuable for understanding the ongoing evolution and spread of this influenza subtype. Our nomenclature system will improve communication among international and local organizations and laboratories working on A/H9 viruses. The use of our system will enhance the global prevention and control capacity of A/H9 AIVs outbreaks. 

Appendix 1 Additional information about proposal for a global classification and nomenclature system for A/H9 influenza viruses.

Appendix 2 Additional information about the H9-HA sequences selected for a global classification and nomenclature system for A/H9 influenza viruses.

Appendix 3 Additional information about the pilot datasets of lineages and clades selected for a global harmonized classification and nomenclature system for A/H9 influenza viruses.
