# Supplementary material for: Proposal for a Global Classification and Nomenclature System for A/H9 Influenza Viruses
Source: Emerg Infect Dis. 2024 Aug;30(8):e231176. doi: 10.3201/eid3008.231176 (PMC11286050; doi:10.3201/eid3008.231176)
Supplement: Appendix 1 — Additional information about proposal for a global classification and nomenclature system for A/H9 influenza viruses. [file 23-1176-Techapp-s1.pdf]

Article DOI: https://doi.org/10.3201/eid3008.231176

EID cannot ensure accessibility for supplementary materials supplied by authors. Readers who have difficulty accessing supplementary content should contact the authors for assistance.

Proposal for a Global Classification and Nomenclature System for A/H9 Influenza Viruses

Appendix 1

Methods

Sequence Data and Metadata Preparation

To provide a comprehensive picture of the A/H9 genetic diversity, we generated a dataset of the hemagglutinin gene that included all the H9-HA sequences available on the GISAID (www.epicov.org) and GenBank (www.ncbi.nlm.nih.gov/nucleotide/) public databases (accessed on July 18, 2022). Multiple sequence entries (i.e., sequences obtained from the same sample, deposited multiple times or available in both databases) or sequences obtained from laboratory-derived viruses were discarded. Sequences were further filtered for length and quality. Specifically, all the sequences with more than 5 ambiguous bases and with a length <1275 bp (75% of the coding region) were removed. If no sequences matching these criteria were available for a specific country and collection year, sequences with a length >900pb were accepted to have the most exhaustive dataset as possible in terms of sequence representativeness and quality.

Sequences alignment, obtained using MAFFT v7.0 (*1*), was manually curated and nucleotides outside the coding region of the mature HA gene were trimmed. After removing sequences containing out-of-frame indels, a preliminary Maximum Likelihood (ML) phylogenetic tree using IQ-TREE v1.6 (*2*) was generated from this dataset to test 'clocklikeness'

of the dated-tip phylogeny using TempEst (*3*). A good correlation between the collection dates (year) of the virus and the divergence from the root was observed (r = 0.82). This analysis helped us to identify outlier sequences, which may be due to mislabeling of the virus (incorrect year of collection), sample contamination by an older virus or sequencing errors. All the outlier sequences were removed from the dataset. In the process, early strains such as A/turkey/Wisconsin/1/1966 were removed.

Furthermore, only the oldest sequence was kept among sequences with 100% identity that were collected in the same country. Finally, since mosaic influenza genome segments have previously been described as resulting from laboratory contamination or artifacts, or from a natural homologous recombination (*4*), the dataset was screened for mosaic structures using the RDP, Geneconv, Maxchi, BootScan, 3Seq and Chimaera methods available in the RDP package v.4 (*5*), applying default settings. The Simplot program v.3.5 was also used to define the locations of recombination breakpoints (*6*). The potential mosaic sequences identified by at least two methods with $p < 1\times10^{-10}$ were considered unreliable and were removed from the dataset. A final dataset (Complete dataset) containing 10,638 HA sequences and the related information, including accession numbers, were produced after the quality check process (Appendix 1 Table 1).

Testing and Selection of PhyCLIP Parameters

PhyCLIP utilizes an integer linear programming (ILP) approach that optimally delineates a tree into statistically principled clusters (*7*), to optimize our clustering results, we tested a range of values for three key parameters: 1) minimum number of sequences (S) that can be quantified as a cluster (S = 3, 5, 10), 2) false discovery rate (FDR) used to infer that the diversity observed for every combinatorial pair of output clusters is significantly distinct from one another (FDR range from 0.1 to 0.2 in increments of 0.05), and 3) multiple of deviations (γ) from the grand median of the mean pairwise sequence patristic distance that defines the within-cluster

divergence limit (WCL) (γ range from 1 to 3 in increments of 1). We used the clustering resulting from the optimal parameter (S = 5, FDR = 0.2 and γ = 3) combinations as a reference for the assignment of clades.

References:

1. Katoh K, Standley DM. MAFFT multiple sequence alignment software version 7: improvements in performance and usability. Mol Biol Evol. 2013;30:772–80. PubMed https://doi.org/10.1093/molbev/mst010

2. Nguyen LT, Schmidt HA, von Haeseler A, Minh BQ. IQ-TREE: a fast and effective stochastic algorithm for estimating maximum-likelihood phylogenies. Mol Biol Evol. 2015;32:268–74. PubMed https://doi.org/10.1093/molbev/msu300

3. Rambaut A, Lam TT, Max Carvalho L, Pybus OG. Exploring the temporal structure of heterochronous sequences using TempEst (formerly Path-O-Gen). Virus Evol. 2016;2:vew007. PubMed https://doi.org/10.1093/ve/vew007

4. Lam TT, Chong YL, Shi M, Hon CC, Li J, Martin DP, et al. Systematic phylogenetic analysis of influenza A virus reveals many novel mosaic genome segments. Infect Genet Evol. 2013;18:367–78. **PMID: 23548803**

5. Martin DP, Lemey P, Lott M, Moulton V, Posada D, Lefeuvre P. RDP3: a flexible and fast computer program for analyzing recombination. Bioinformatics. 2010;26:2462–3. PubMed https://doi.org/10.1093/bioinformatics/btq467

6. Lole KS, Bollinger RC, Paranjape RS, Gadkari D, Kulkarni SS, Novak NG, et al. Full-length human immunodeficiency virus type 1 genomes from subtype C-infected seroconverters in India, with evidence of intersubtype recombination. J Virol. 1999;73:152–60. PubMed https://doi.org/10.1128/JVI.73.1.152-160.1999

7. Han AX, Parker E, Scholer F, Maurer-Stroh S, Russell CA. Phylogenetic clustering by linear integer programming (PhyCLIP). Mol Biol Evol. 2019;36:1580–95. PubMed https://doi.org/10.1093/molbev/msz053

8. Sagulenko P, Puller V, Neher RA. TreeTime: Maximum-likelihood phylodynamic analysis. Virus Evol. 2018;4:vex042. PubMed https://doi.org/10.1093/ve/vex042

9. Hadfield J, Megill C, Bell SM, Huddleston J, Potter B, Callender C, et al. Nextstrain: real-time tracking of pathogen evolution. Bioinformatics. 2018;34:4121–3. PubMed https://doi.org/10.1093/bioinformatics/bty407

10. Carnaccini S, Perez DR. H9 influenza viruses: an emerging challenge. Cold Spring Harb Perspect Med. 2020;10:a038588. PubMed https://doi.org/10.1101/cshperspect.a038588

11. Guan Y, Shortridge KF, Krauss S, Webster RG. Molecular characterization of H9N2 influenza viruses: were they the donors of the "internal" genes of H5N1 viruses in Hong Kong? Proc Natl Acad Sci U S A. 1999;96:9363–7. PubMed https://doi.org/10.1073/pnas.96.16.9363

12. Liu S, Ji K, Chen J, Tai D, Jiang W, Hou G, et al. Panorama phylogenetic diversity and distribution of Type A influenza virus. PLoS One. 2009;4:e5022. PubMed https://doi.org/10.1371/journal.pone.0005022

13. Fusaro A, Monne I, Salviato A, Valastro V, Schivo A, Amarin NM, et al. Phylogeography and evolutionary history of reassortant H9N2 viruses with potential human health implications. J Virol. 2011;85:8413–21. PubMed https://doi.org/10.1128/JVI.00219-11

14. Dalby AR, Iqbal M. A global phylogenetic analysis in order to determine the host species and geography dependent features present in the evolution of avian H9N2 influenza hemagglutinin. PeerJ. 2014;2:e655. PubMed https://doi.org/10.7717/peerj.655

15. Li C, Wang S, Bing G, Carter RA, Wang Z, Wang J, et al. Genetic evolution of influenza H9N2 viruses isolated from various hosts in China from 1994 to 2013. Emerg Microbes Infect. 2017;6:e106. PubMed https://doi.org/10.1038/emi.2017.94

16. Zhuang Q, Wang S, Liu S, Hou G, Li J, Jiang W, et al. Diversity and distribution of type A influenza viruses: an updated panorama analysis based on protein sequences. Virol J. 2019;16:85. PubMed https://doi.org/10.1186/s12985-019-1188-7

Appendix 1 Table 1. Ultra-fast bootstrap (UFB) values, standard bootstraps (SB) and SH-like supports (aLRT SH-like) obtained for each clade nodes from the analyses of different datasets (complete and pilot datasets) using different software (IQ-TREE and PhyML).

**Dataset**		**Complete datasets**		**Pilot datasets**	
**Software**		**IQ-TREE**	**PhyML**	**IQ-TREE**	**IQ-TREE**
**Lineage**	**Clades**	**UFB**	**aLRT SH-like**	**UFB**	**SB**
G	G1	100	1	100	100
	G2	100	0.997	100	99
	G3	100	0.999	100	100
	G4	100	0.996	100	100
	G5.1	99	1	100	100
	G5.2	99	0.733	100	96
	G5.3.1	100	0.999	100	100
	G5.3.2	100	1	100	93
	G5.4	95	1	100	100
	G5.5	100	0.992	99	100
	G5.6	100	1	100	100
	G5.7	100	0.995	85	66
Y	Y1	100	1	100	100
	Y2.1	100	0.967	99	100
	Y2.2	100	1	100	100
	Y3	96	0.85	100	100
	Y4	100	1	100	100
	Y5	100	0.852	99	92
	Y6	100	1	100	100
	Y7	84	0.868	97	77
	Y8	100	1	100	100
	Y9	100	1	100	100

Dataset		Complete datasets		Pilot datasets	
Software		IQ-TREE	PhyML	IQ-TREE	IQ-TREE
Lineage	Clades	UFB	aLRT SH-like	UFB	SB
B	B1	100	0.959	99	86
	B2	94	0.91	99	96
	B3	81	0.927	99	91
	B4.1	100	0.998	100	98
	B4.2	100	0.912	97	60
	B4.3	100	0.925	100	99
	B4.4	93	0.988	100	100
	B4.5	91	0.908	99	90
	B4.6	82	0.92	97	73
	B4.7	82	0.952	100	100

Appendix 1 Table 2. Representative amino acid residues of each clade.

Lineage	Clade	Amino acid mutations based on ancestral reconstruction using TreeTime *(8, 9)*
Y	Y1	V288I, V317A, I451V, R479K
	Y2	N45D, F104L, N109R, Q112K, V153F, N161T, T182N, I249V, K276R, V288I, D319N, R358K, V365I, K487R
	Y3	V153F, N267D, V352T
	Y4	T54K, E72T, H146Q, V153I, N264K, R320K, V365I, E501D, K505R
	Y5	S103A, N398S
	Y6	K131A, H146Q, V153F, D155N, E162N, N165S, A317V, D319G, E363V, N398S, I451M
	Y7	V269I
	Y8	I451R
	Y9	I20V, N94R, N109R, Q112K, I114L, Q115L, I116L, T120R, I121T, V153I, N161D, E162W, T182I, V194I, D319G, N455K, Q480L, Q483K, G502E, L527M
	Y2.1	L69I, I166V, V206M, V302A
	Y2.2	K164E, E459D, F523L
G	G1	S83P, V95I, G135D, T195A, I202V, V213A, V249I, N264K, V300I, N374S, V393I
	G2	G135D, D178E, S370T
	G3	N264T, V411I

Lineage	Clade	Amino acid mutations based on ancestral reconstruction using TreeTime *(8, 9)*
	G4	H34Q, A132S, S165N, E180D, N183T, D198E, L216Q, R301K
	G5	A108S, A132S, S140N, S148N, I186T, D198N, N200D, M206L
	G5.1	N148S, L150F, T182R, T186I, L216Q, I217T, V226I, I288V, I365V
	G5.2	S486A
	G5.3	S150L, N165D
	G5.4	R317K, N374T
	G5.5	K483T
	G5.6	L150V
	G5.7	D262N, T295N, V496I
	G5.3.1	Q112R, R162Q, Q467H, L488I
	G5.3.2	V24I, H28Q, H48R, S108A, T120A, T127D, D135N, V153I, V169I, R317K, D359G, I365V, V376I, K381R
B	B1	N395A
	B2	I153V
	B3	N148S
	B4	N264K, V269A
	B4.1	T395N
	B4.2	K481R
	B4.3	K492R
	B4.4	S125T
	B4.5	D221N, R236K
	B4.6	D221N
	B4.7	D135G, E163G

Note: all HA positions follow the H9 numbering. The red color indicates that these sites are associated with host tropism, virulence or identified antigenic sites (*10*).

Appendix 1 Table 3. Comparison of previous and current nomenclature systems.

**Previous studies**			**Current study**
**Publication**	**Clade classification and nomenclature**		**Clade classification and nomenclature**
Guan Y et al. (1999) (*11*)	G1		G1-G5
	BJ94(Y280)		B1-B4
	Y439		Y4-Y9
	TY66		Y1-Y3
Liu S et al. (2009) (*12*)	h9.1, h9.2		Y1-Y3
	h9.3		Y4-Y9
	h9.4	h9.4.1	G1-G5
		h9.4.2	B1-B4
Fusaro A et al. (2011) (*13*)	G1-A		G1
	G1-B		G5
	G1-C		G-like
	G1-D		G2
Dalby AR et al. (2014) (*14*)	Clade A		Y1-Y9
	Clade B		G1-G5
	Clade C, Main Chinese Clade		B1-B4
Li C et al. (2017) (*15*)	0–15	0, 5–7, 9–11, 13	B-like
		1	Y-like
		2	Y3, Y8
		3	G3
		4	G4
		8	B1
		12	B2
		14	B3
		15	B4
Zhuang Q et al. (2019) (*16*)	H9.1		Y1-Y9
	H9.2a		G1-G5
	H9.2b		B1-B4
Carnaccini S et al. (2020) (10)	h9.1 (h9.1.2)		Y1-Y3
	Y439-h9.2 (Korea h9.2.2)		Y4-Y9
	BJ94-h9.3		B1-B4
	G1-h9.4.1 Eastern		G1-G4
	G1-h9.4.2 Western		G5

Appendix 1 Table 4. Temporal, spatial and host distribution characteristics of lineage Y.

Lineage/Clade	Time range	Countries of origin	Type of host	Number of taxa	The earliest strain
Y	1976–2021	Argentina, Australia, Austria, Bangladesh, Belgium, Cambodia, Canada, Chile, China, Egypt, Finland, France, Georgia, Germany, Hungary, Iran, Ireland, Italy, Japan, Malaysia, Mexico, Mongolia, Netherlands, New Zealand, Norway, Poland, Portugal, Russian Federation, Singapore, South Africa, South Korea, Sweden, Switzerland, Thailand, Ukraine, United Kingdom, United States, Vietnam, Zambia	Avian, Avian domestic, Avian wild, Environment, Mammalian	622	A_Duck_Hong_Kong_86_1976
Y1	2000–2016	Mexico, United States	Avian wild, Environment	39	A_shorebird_Delaware_Bay_277_2000
Y2	1993–2017	Argentina, Chile, United States	Avian domestic, Avian wild, Environment	13	A_Quail_Arkansas_29209–1_1993
Y3	1976–2020	Canada, China, Hungary, Italy, New Zealand, South Korea, United States	Avian domestic, Avian wild, Environment	123	A_Duck_Hong_Kong_86_1976
Y4	2010–2019	China, Georgia, Singapore, United Kingdom, United States	Avian domestic, Avian wild	22	A_chicken_England_1415–51184_2010
Y5	2003–2007	United States	Avian wild	42	A_ruddy_turnstone_Delaware_1016406_2003
Y6	2009–2018	Cambodia, Vietnam	Avian domestic, Avian wild	6	A_duck_Vietnam_OIE-2313_2009
Y7	1993–2010	Finland, Germany, Ireland, Italy, Netherlands, Sweden, United Kingdom, United States	Avian domestic, Avian wild	26	A_mallard_Ireland_PV46B_1993

Lineage/Clade	Time range	Countries of origin	Type of host	Number of taxa	The earliest strain
Y8	1995–2021	Australia, Austria, Bangladesh, Belgium, China, Finland, France, Germany, Iran, Italy, Japan, Mongolia, Netherlands, Norway, Poland, Portugal, Russian Federation, South Africa, South Korea, Sweden, Switzerland, Thailand, Ukraine, United Kingdom, United States, Vietnam, Zambia	Avian, Avian domestic, Avian wild, Environment	154	A_ostrich_South_Africa_9508103_1995
Y9	1996–2018	Egypt, Malaysia, South Korea	Avian, Avian domestic, Avian wild, Environment, Mammalian	186	A_chicken_Korea_25232–96006_1996
Y-like	1978–2001	China, Japan, Malaysia	Avian domestic	11	A_Duck_Hong_Kong_366_1978
Y2.1	1993–1996	United States	Avian domestic Avian wild	6	A_Quail_Arkansas_29209–1_1993
Y2.2	2007–2017	Argentina, Chile	Avian wild Environment	7	A_rosy-billed_pochard_Argentina_CIP051–559_2007

Appendix 1 Table 5. Temporal, spatial and host distribution characteristics of lineage G.

Lineage/Clade	Time range	Countries of origin	Type of host	Number of taxa	The earliest strain
G	1997–2022	Afghanistan, Algeria, Bangladesh, Benin, Burkina Faso, China, Egypt, Germany, Ghana, India, Iran, Iraq, Israel, Japan, Jordan, Kenya, Kuwait, Lebanon, Libya, Morocco, Nepal, Nigeria, Oman, Pakistan, Qatar, Russian Federation, Saudi Arabia, Senegal, Togo, Tunisia, Uganda, United Arab Emirates, Vietnam	Avian, Avian domestic, Avian wild, Environment, Human	1643	A_quail_Hong_Kong_G1_1997
G1	2003–2007	Israel, Jordan, Lebanon	Avian, Avian domestic	43	A_chicken_Jordan_12_2003
G2	1999–2005	Iran	Avian domestic, Avian wild	21	A_chicken_Iran_705_1999
G3	2000–2004	China	Avian domestic, Avian wild	29	A_quail_Shantou_782_2000
G4	1997–2017	China, Vietnam	Avian, Avian domestic, Avian wild, Environment, Human	74	A_quail_Hong_Kong_G1_1997
G5	1998–2022	Afghanistan, Algeria, Bangladesh, Benin, Burkina Faso, Egypt, Ghana, India, Iran, Iraq, Israel, Jordan, Kenya, Kuwait, Lebanon, Libya, Morocco, Nepal, Nigeria, Oman, Pakistan, Qatar, Russian Federation, Saudi Arabia, Senegal, Togo, Tunisia, Uganda, United Arab Emirates	Avian, Avian domestic, Avian wild, Environment, Human	1427	A_chicken_Iran_725_1998
G-like	1997–2007	Germany, Iran, Israel, Japan, Pakistan, Saudi Arabia, United Arab Emirates	Avian, Avian domestic	49	A_parakeet_Chiba_1_1997
G5.1	2005–2011	United Arab Emirates	Avian, Avian domestic, Avian wild	10	A_white_bellied_bustard_United_Arab_Emirates_1127_1_2005

Lineage/Clade	Time range	Countries of origin	Type of host	Number of taxa	The earliest strain
G5.2	1998–2009	Iran, Iraq, United Arab Emirates	Avian, Avian domestic	35	A_chicken_Iran_725_1998
G5.3	2007–2022	Afghanistan, India, Iran, Iraq, Nepal, Pakistan	Avian, Avian domestic, Avian wild, Human	146	A_Chicken_Nepal_JHAPA_28_2007
G5.4	2000–2013	Afghanistan, Iran, Pakistan, Saudi Arabia	Avian domestic	35	A_chicken_Saudi_Arabia_2525_2000
G5.5	2006–2021	Algeria, Benin, Burkina Faso, Ghana, Israel, Jordan, Kenya, Libya, Morocco, Nigeria, Oman, Qatar, Saudi Arabia, Senegal, Togo, Tunisia, Uganda, United Arab Emirates	Avian, Avian domestic, Avian wild, Environment, Human	331	A_avian_Libya_RV35D_2006
G5.6	2006–2021	Egypt, Israel, Jordan, Lebanon, Russian Federation	Avian, Avian domestic	497	A_chicken_Israel_1638_2006
G5.7	2003–2022	Bangladesh, India, Kuwait, Pakistan	Avian, Avian domestic, Avian wild, Environment, Human	332	A_chicken_Chandigarh_2048_2003
G5.3.1	2007–2013	India, Nepal	Avian domestic	18	A_Chicken_Nepal_JHAPA_28_2007
G5.3.2	2008–2022	Afghanistan, Iran, Iraq, Pakistan	Avian, Avian domestic, Avian wild, Human	128	A_chicken_Afghanistan_329–6vir09-AFG-Khost9_2008

Appendix 1 Table 6. Temporal, spatial and host distribution characteristics of lineage B.

Lineage/Clade	Time range	Countries of origin	Type of host	Number of taxa	The earliest strain
B	1994–2021	Cambodia, China, Indonesia, Japan, Laos, Malaysia, Myanmar, Russian Federation, South Korea, Tajikistan, Vietnam	Avian, Avian domestic, Avian wild, Environment, Human, Mammalian	8373	A_chicken_Beijing_1_1994
B1	1997–2013	China, Japan	Avian, Avian domestic, Avian wild, Environment, Mammalian	108	A_Chicken_Sichuan_5_1997
B2	1996–2016	China, Japan, Vietnam	Avian, Avian domestic, Avian wild, Human, Mammalian	425	A_Quail_Shanghai_8_1996
B3	1998–2017	China, Japan	Avian, Avian domestic, Human, Mammalian	51	A_Shaoguan_408_1998
B4	1999–2021	Cambodia, China, Indonesia, Japan, Laos, Malaysia, Myanmar, Russian Federation, South Korea, Tajikistan, Vietnam	Avian, Avian domestic, Avian wild, Environment, Human, Mammalian	7606	A_chicken_Shandong_JN_1999
B-like	1994–2017	China, Japan	Avian, Avian domestic, Avian wild, Human, Mammalian	183	A_chicken_Beijing_1_1994
B4.1	2000–2005	China	Avian domestic, Avian wild	59	A_partridge_Shantou_5692_2000
B4.2	2003–2014	China, Vietnam	Avian, Avian domestic, Avian wild, Environment, Human, Mammalian	77	A_chicken_Guangdong_B7_2003
B4.3	2002–2013	China	Avian domestic	163	A_chicken_Guangdong_A7_2002
B4.4	2009–2020	China	Avian, Avian domestic, Mammalian	205	A_Duck_Fujian_1753_2009
B4.5	2011–2020	Cambodia, China, Indonesia, Japan, Laos, Malaysia, Vietnam	Avian, Avian domestic, Avian wild, Environment, Human, Mammalian	1116	A_chicken_Anhui_A12_2011

Lineage/Clade	Time range	Countries of origin	Type of host	Number of taxa	The earliest strain
B4.6	2012–2020	China, South Korea	Avian, Avian domestic, Avian wild, Environment, Mammalian	882	A_chicken_Shandong_QD6_2012
B4.7	2012–2021	Cambodia, China, Japan, Laos, Myanmar, Russian Federation, Tajikistan, Vietnam	Avian, Avian domestic, Avian wild, Environment, Human, Mammalian	4280	A_chicken_Anhui_A225_2012

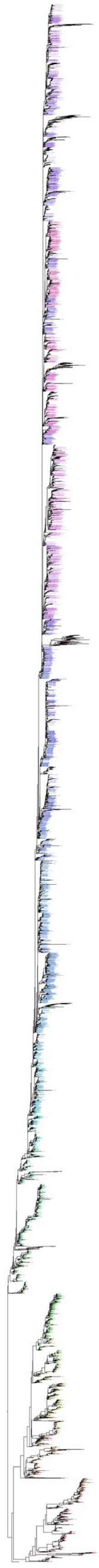

Appendix 1 Figure 1. Clustering based on optimal parameters of PhyCLIP. The numbers on the tree indicate the clade classification of PhyCLIP.

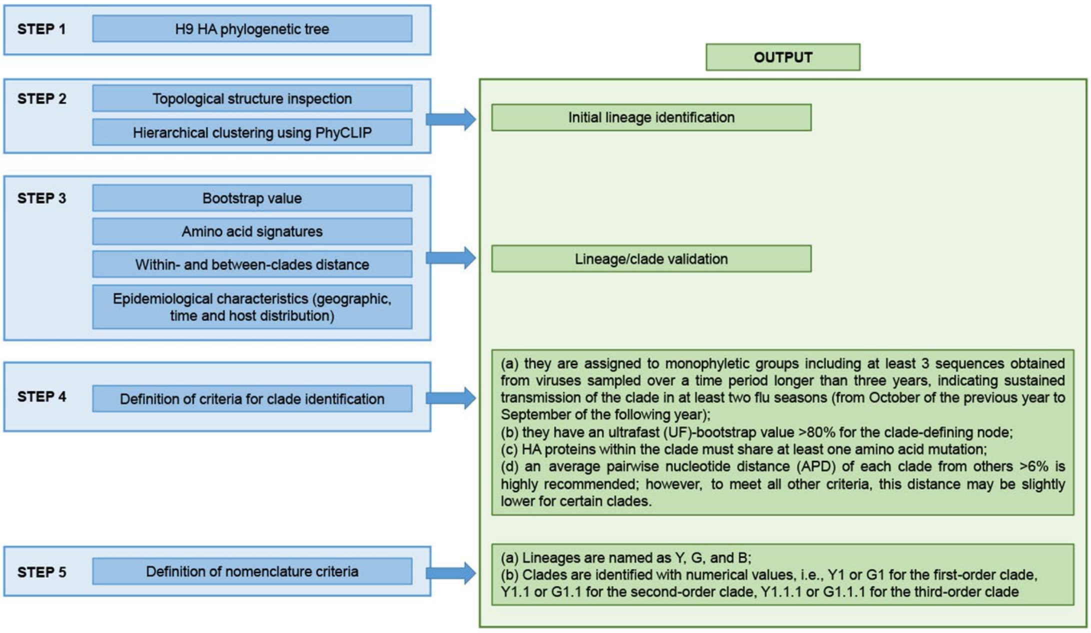

Appendix 1 Figure 2. Scheme of the strategy adopted to classify A/H9 hemagglutinin sequences into lineages and clades.

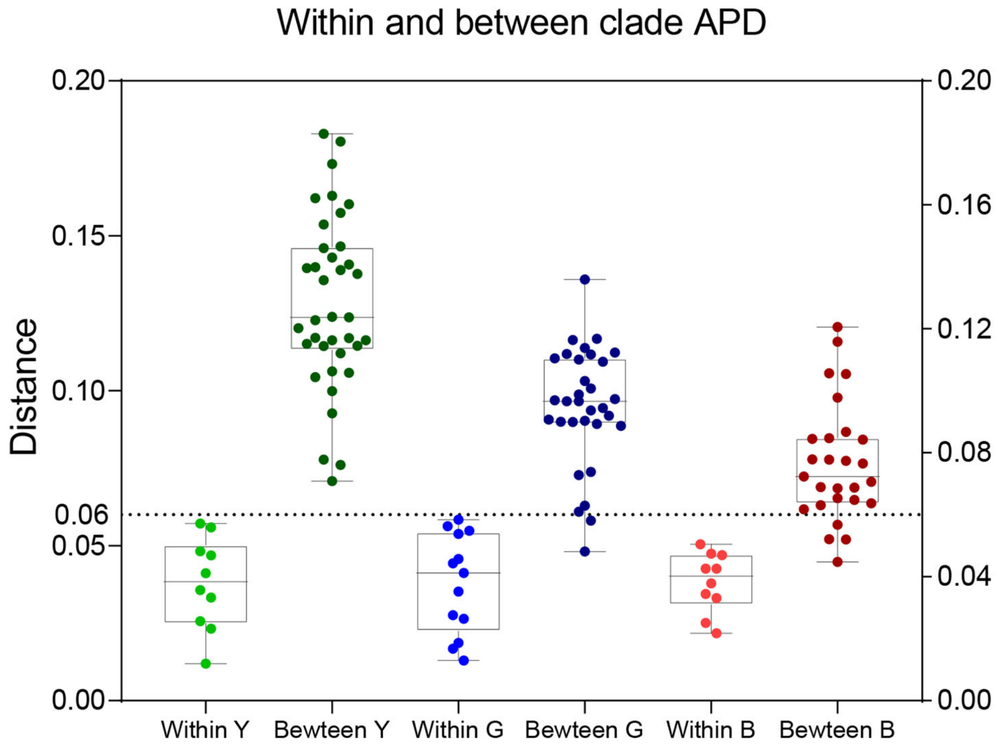

Appendix 1 Figure 3. Average pairwise distances within- and between-clades of A/H9 influenza viruses. The boxplot displays the average pairwise distances (APD) calculated within and between each clade of the three lineages.

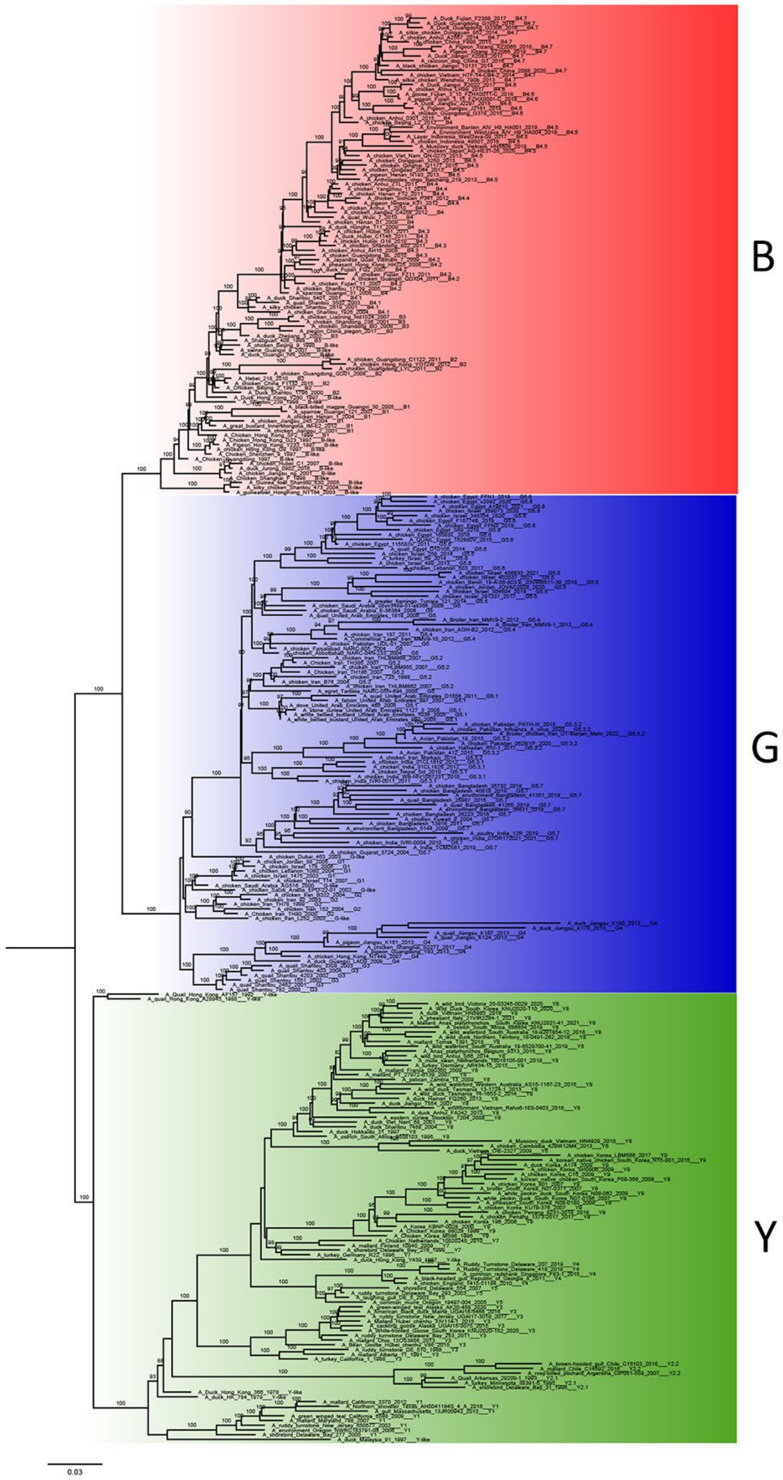

Appendix 1 Figure 4. Pilot Maximum likelihood phylogenetic trees of the H9-HA gene sequences obtained by using the complete small representative dataset available in Appendix 3 for all 3 lineages (Appendix 3 “Pilot Complete Genomes”).
